# A hyper-thermostable α-amylase from *Pyrococcus furiosus* accumulates in *Nicotiana tabacum* as functional aggregates

**DOI:** 10.1186/s12896-017-0372-3

**Published:** 2017-06-19

**Authors:** Hong Zhu, L. Bruce Reynolds, Rima Menassa

**Affiliations:** 1 Agriculture and Agri-Food Canada, London Research and Development Centre, London, Ontario Canada; 2 Department of Biology, University of Western Ontario, London, Ontario Canada

**Keywords:** Amylase, Thermostable, Tobacco, Hyperthermostable enzyme, PFA, Starch hydrolase, Molecular farming, Recombinant protein production, Transgenic plants

## Abstract

**Background:**

Alpha amylase hydrolyzes α-bonds of polysaccharides such as starch and produces malto-oligosaccharides. Its starch saccharification applications make it an essential enzyme in the textile, food and brewing industries. Commercially available α-amylase is mostly produced from *Bacillus* or *Aspergillus*. A hyper-thermostable and Ca ^2++^ independent α-amylase from *Pyrococcus furiosus* (PFA) expressed in *E.coli* forms insoluble inclusion bodies and thus is not feasible for industrial applications.

**Results:**

We expressed PFA in *Nicotiana tabacum* and found that plant-produced PFA forms functional aggregates with an accumulation level up to 3.4 g/kg FW (fresh weight) in field conditions. The aggregates are functional without requiring refolding and therefore have potential to be applied as homogenized plant tissue without extraction or purification. PFA can also be extracted from plant tissue upon dissolution in a mild reducing buffer containing SDS. Like the enzyme produced in *P. furiosus* and in *E. coli*, plant produced PFA preserves hyper-thermophilicity and hyper-thermostability and has a long shelf life when stored in lyophilized leaf tissue. With tobacco’s large biomass and high yield, hyper-thermostable α-amylase was produced at a scale of 42 kg per hectare.

**Conclusions:**

Tobacco may be a suitable bioreactor for industrial production of active hyperthermostable alpha amylase.

**Electronic supplementary material:**

The online version of this article (doi:10.1186/s12896-017-0372-3) contains supplementary material, which is available to authorized users.

## Background

Alpha amylase is an enzyme with widespread applications in food, textile, detergent and alcohol production and other industries. In the 1990s, enzymes from an archaeon *Pyrococcus furiosus* were identified, which are valuable due to their hyper-thermostability in multiple applications [1–5]. Among these enzymes, an extracellular α-amylase, *P.furiosus* α-amylase (PFA), was cloned, characterized and expressed in *E.coli* by Dong et al. [6]. PFA's advantages over α-amylases from other sources include an optimal temperature of 100 °C, a half-life of 13 h at 98 °C, and Ca^2+^-independent functionality. PFA is very desirable in industrial applications, but its use is hampered by the high production cost in *E.coli* because it accumulates in the form of insoluble inclusion bodies, and a tedious solubilization procedure is required to produce a soluble functional enzyme [7]. Over the years, there has been interest in exploring new strategies to increase the solubility and yield of recombinant PFA. For example, about 28 mg (109,000 U) of soluble PFA is produced in 1 litre of culture by co-expression of thioredoxin and induction at 18 °C with 1% ethanol [6]. Recently, to test if molecular chaperones have important roles in protein folding in *P.furiosus*, Peng and co-workers [8] co-expressed PFA with chaperones from *P. furiosus* in *E.coli*. They reported that both chaperonin and a small heat shock protein (sHSP) increased the solubility of PFA to a certain degree, while prefoldin seemed to be the most efficient, and increased the enzyme activity of the supernatant to about 60,000 U/g wet weight from about 5000 U/g wet weight without chaperone, and a visible band in the supernatant was detected in a Coomassie stained gel. Adding chaperonin or sHSP to prefoldin in co-expression experiments did not further improve the solubility of recombinant PFA. By cloning prefoldin and PFA into one plasmid, they further increased the solubility of *E.coli*-made PFA to about 50%, up from unquantifiable levels on a Coomassie stained gel, and the enzyme activity reached about 84,000 U/g wet weight. When they co-expressed prefoldin in a high copy plasmid and PFA in a low copy plasmid to increase the ratio of prefoldin to PFA, they almost eliminated insoluble PFA, but the total amount of recombinant PFA was reduced [8]. Wang et al. [9] produced soluble PFA by expressing PFA in *Bacillus amyloliquefaciens*, the organism currently used in industry to produce amylase (BAA). They modified the PFA gene by mimicking expression and secretion elements such as codon usage bias, mRNA structure, and promoter elements of BAA. PFA was successfully expressed in *B. amyloliquefaciens* and secreted into the medium. The yield of PFA was 2,000 U/ml of supernatant and 2,714 U/ml of total culture. The *B. amyloliquefaciens*-made PFA has similar temperature and pH optima as the native- and *E.coli*-made PFA [9]. Using plants to synthesize recombinant proteins has increasingly become an alternative for low cost medical materials, pharmaceuticals, as well as industrial purposes [10–15]. Among the plants used, tobacco has the benefit of being a non-food and non-feed crop, and providing large amounts of leaf biomass that can be harvested before flowering thus limiting spread of the transgene through pollen or seeds. Because of this, tobacco is considered a safe platform in molecular farming [16].

In our study, we found that a thermostable α-amylase from *P. furiosus* (PFA) accumulates in leaves as insoluble but active aggregates that do not require refolding, and that are active in harsh denaturing conditions. This indicates that plants have the ability to store recombinant proteins as aggregates and therefore keep them out of their biological pathways. This is an advantage that we can exploit in molecular farming.

## Results and Discussion

### Transient expression of *PFA* in tobacco

**Fig. 1:**
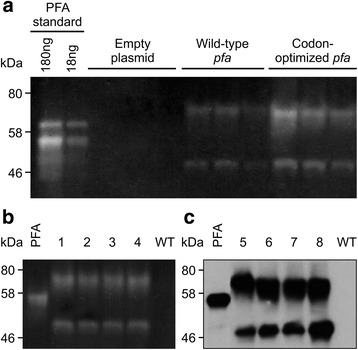
PFA accumulates in *Nicotiana tabacum* in both transient expression and stable transformation. **a** Codon optimization improves accumulation of PFA in transient expression. Zymogram with tobacco leaf extracts transiently expressing *pfa* or an empty expression vector. Three replicates from three different plants are shown for each treatment. The bright bands indicate areas where starch is degraded by PFA. Each lane was loaded with 20 μl plant extract from 0.1 g leaf material extracted with 400 μl protein extraction buffer. PAGE-purified *E.coli-* produced PFA was used as standard. **b** Zymogram and (**c**) western blot hybridization analysis with anti-PFA antibodies of stable transgenic tobacco lines. PFA: 40 ng of *E. coli*-produced purified PFA. Lanes 1-8 represent eight independent transgenic lines from *N. tabacum cv*. TI95 (1-4 and 7-8) and I64 (5-6). The tobacco lines shown are representative of 16 *N. tabacum* cultivars used for producing 400 individual lines (25 lines/cultivar). WT: wild type, untransformed tobacco plant. Each lane was loaded with 5 μg total protein extracted with reducing extraction buffer

**Fig. 2:**
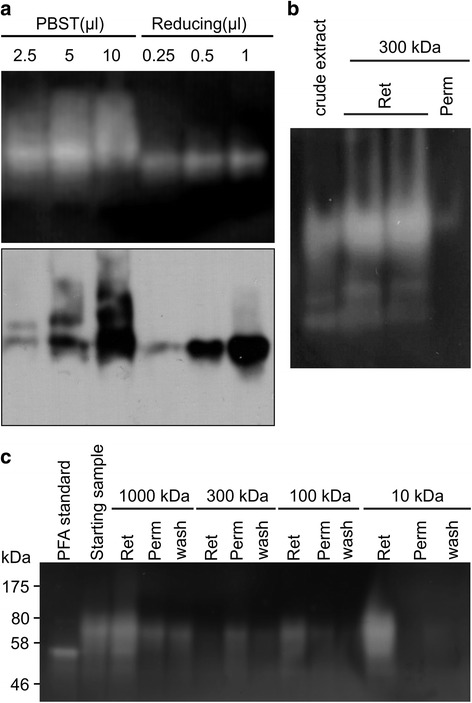
In native conditions, PFA forms aggregates. **a**. Zymogram (top panel) and western hybridization analysis with anti-PFA antibodies (*lower panel*) of plant-produced recombinant PFA from a stable transgenic plant on native PAGE. PBST: recombinant PFA extracted with PBST-based extraction buffer. Reducing: recombinant PFA extracted with reducing extraction buffer. μl: the amount of plant extract loaded onto each lane. Plant proteins were extracted from 20 mg of powdered freeze- dried whole leaf with 400 μl extraction buffer. Ten times more extract from PBST-based extraction was loaded onto the gel due to expected lower PFA extraction in this buffer. The zymogram does not show a difference in band intensities associated with loading volumes for either extraction method because the lowest loading volume contained enough PFA for digesting the starch present in the gel. **b** Crude extract in non-reducing buffer was filtered through a tangential flow filtration Pellicon XL cassette with 300 kDa cut-off membrane. lower functional PFA extraction in this buffer. **c** Crude extract in reducing buffer (80 ml) was sequentially filtered through Pellicon XL cassettes with 1000, 300, 100 and 10 kDa cut-off membranes. The membrane was washed with 20 ml buffer (wash). The permeate (Perm) and wash from one membrane were pooled and applied onto the next size cut-off membrane. The retentate (Ret) was about 8 ml in each case. Twenty μl of sample from each fraction were loaded onto each lane, and proteins were separated by SDS-PAGE

The gene coding PFA *pfa* was cloned in a plant expression vector pCaMterX [17] with an N-terminal plant signal peptide and a C-terminal ER (endoplasmic reticulum) retrieval peptide to target the protein to the secretory pathway and retrieve it to the ER. We chose to target the protein to the ER because it has consistently been shown to provide a superior environment for folding and storing recombinant proteins in plant cells, while cytosolic expression results in little to no protein accumulation in the majority of the cases [11, 16, 18], reviewed in [19]. To determine if PFA would accumulate in plants, the PFA protein was produced in tobacco leaves via *Agrobacterium*-mediated transient expression. Total soluble protein was extracted from leaves and separated by SDS (sodium dodecyl sulfate)-PAGE (polyacrylamide gel electrophoresis). PFA enzymatic activity was determined via starch degradation analysis on a zymogram and PAGE purified *E.coli*-produced PFA was used as standard (Fig. 1a). The zymogram clearly shows that functional PFA was produced in tobacco leaves and that it is able to degrade starch even in denaturing conditions. Plant-synthesized PFA (calculated size of 50 kDa) appears as two major bands about 50 and 70 kDa. Dong et al. [6] observed a 44 kDa and a 66 kDa band in *E.coli* **-**produced PFA. Both bands are smaller than the expected monomer and dimer sizes of 52 kDa and 104 kDa, and the authors suggested that PFA monomers and homodimers may be partially folded in the polyacrylamide gel, resulting in a smaller apparent size than their expected size [6]. This behavior was also observed with other enzymes from the hyperthermophilic archaea *P. furiosus* and *P. woesie* [2, 4, 20]. Savchenko et al reported that this enzyme is monomeric because it belongs to glycosyl hydrolases' family13 which contains only monomeric enzymes. Their evidence includes mass spectrometry showing a molecular mass of 50 kDa, and light scattering measurement indicating 48 kDa molecular size. They suggested that the dimeric-sized band is due to partially denatured PFA reacting with another PFA monomer to form a disulfide bridge in the denaturing conditions of SDS PAGE [21]. We believe the same could be true in the case of plant-made PFA, and that the observed bands represent PFA monomer and dimer formed in the denaturing conditions of SDS-PAGE. Our *E.coli*-expressed PFA however appears as only monomeric in SDS PAGE possibly due to additional residues consisting of Trx, His, S Tags and thrombin cleavage site, totalling about 17.5 kDa, introduced by the expression vector pET32. The *E.coli*-produced PFA appears as a double band in Fig. 1a, but as one band in Fig. 1b and c, Fig. 2c, and Fig. 4a. We have seen this behavior throughout our experiments and we suspect this may be due to partial folding of this protein in Fig. 1a. Codon optimization of prokaryotic codon usage to match the eukaryotic hosts' codon usage has often been used as a strategy to increase accumulation of recombinant proteins [22, 23]. Since *P. furiosus* is an archaeon, *pfa* was codon-optimized to match tobacco codon usage [14]. Upon transient expression in tobacco, we found that codon-optimization increases PFA accumulation by more than threefold, from 2.24 ± 1.12 ng/mg F.W. to 7.79 ± 1.98 ng/mg FW (Fig. 1a).

### Creation of stable transgenic tobacco lines

Although transient expression is a useful tool for determining the best construct for expression and for quick production of small amounts of protein for characterization, stable transgenic lines are required for industrial production of kilogram amounts of industrial enzymes such as α-amylase. Therefore, the codon-optimized *pfa* gene was transformed into tobacco via *Agrobacterium*-mediated transformation and 400 independent transgenic lines from 16 *Nicotiana* cultivars expressing PFA were created [12]. Zymogram analysis was conducted on all 400 lines, and all lines express functional PFA. A representative zymogram of four independent lines and a western blot of another four independent transgenic lines from *N. tabacum* cv TI95 and I64 are shown in Fig. 1b and c. Densitometric analysis of Fig. 1b against a dilution series of known amounts of *E. coli*-produced PFA standard indicates PFA accumulation in lines 1–4 of 1.05 ± 0.33% of total leaf protein.

### PFA accumulates as aggregates in plants

To determine if native PFA is present in plants in its monomeric form, leaf tissue was extracted in non-reducing buffer or in reducing buffer and proteins were separated by native PAGE and analyzed by both zymogram and western hybridization. Upon extraction in reducing conditions, one major band is evident on the native gels in both zymogram and western hybridization (Fig. 2a). Although both monomer and dimer are observed by denaturing SDS-PAGE (Fig. 1), only one band appears in native PAGE Fig. 2a), indicating that the enzyme is present in only one form, likely its monomeric form. Upon extraction in non-reducing conditions with PBS-Tween-based buffer, western hybridization and zymogram analysis revealed the presence of a higher molecular weight smear, likely consisting of PFA aggregates. The smear was not observed upon extraction in reducing buffer in the presence of SDS, an indication that aggregates were dissociated in reducing conditions (Fig. 2a, lower panel). The fact that aggregates are actively degrading starch on zymograms indicates that they do not require to be dissociated for activity (Fig. 2a, upper panel).

In an attempt to purify PFA from plant extracts by tangential flow filtration in native conditions, we saw that most PFA was retained by a membrane with 300 kDa cut-off pore size which indicates that the PFA aggregates are larger than 300 kDa in native conditions and are enzymatically functional (Fig. 2b). A second experiment was carried out to purify PFA from plant extract in reducing conditions, in which the permeate and wash from one pore size membrane were pooled and subjected to filtration with a membrane of smaller cut-off pore size (sequentially starting at 1000 kDa, then 300, 100, and 10 kDa). The starting sample was heat-treated at 70 °C for 5 min to denature and precipitate other plant proteins and cleared by centrifugation at 4500 x g for 15 min. Although most PFA was recovered and concentrated in the retentate of the 10 kDa cut-off membrane, a significant fraction PFA did not pass through the 1000 kDa cut-off membrane suggesting that PFA forms aggregates of a molecular weight higher than 1000 kDa (Fig. 2c). Some proteins form aggregates after heat treatment [24], but in Fig. 2b large aggregates of more than 300 kDa were also observed in native conditions with no heat treatment. It was previously reported that high accumulation of recombinant proteins in plant chloroplasts leads to the formation of inclusion bodies [25]; PFA accumulates in inclusion bodies in *E. coli*, and requires refolding for activity [7]. Our results suggest that ER-targeted plant-made PFA is correctly folded since aggregates are enzymatically functional, obviating the need for refolding, thus facilitating the process and reducing the cost of PFA production.

### Optimization of reducing extraction buffer

To optimize the reducing extraction buffer, we tested several combinations of DTT and SDS in Tris buffer pH 7.5. When Tris-SDS buffer with or without DTT was used, a similar amount of active PFA was extracted (Additional file 1: Figure S1a), while Tris-DTT required SDS to be present for efficient extraction of PFA (Additional file 1: Figure S1b). SDS concentration of at least 0.6% (w/v) was needed for optimal PFA extraction (Additional file 1: Figure S1c). Although DTT does not play a role in extraction, it appears to stabilize PFA in the extract. The same sample diluted in 10 volumes of Tris-SDS or in Tris-SDS-DTT and incubated for 2.5 h at room temperature showed more residual PFA when DTT was included (Additional file 1: Figure S1d). The results suggest that DTT can either prevent PFA from degradation by proteases or can keep PFA in solution by preventing aggregation. Therefore, the buffer we used to effectively extract active recombinant PFA from transgenic tobacco is 50 mM Tris, 20 mM DTT and 1% SDS. Savchenko and co-workers reported that DTT destabilized PFA at 115 °C and pH 8.5. Cysteine 165 (Cys-165) of PFA is a Zn^2+^ ligand, which is key for the thermostability of this enzyme, and DTT removes Zn^2+^ from the enzyme through its chelating property. However, their work showed that Cys-165 is only accessible when it is partially denatured [21]. In our experiment, the protein extract was incubated at room temperature and therefore the protein was not denatured, and DTT could not remove Zn^2+^; instead, it kept PFA stable in the plant extract. By using this extraction buffer, we found that over ten times more functional PFA can be extracted from plant tissue than using PBS-based buffer. Densitometric quantification of bands in the zymogram (Additional file 1: Figure S1a) indicates that the accumulation of active PFA in stable transgenic tobacco is 66.3 ± 9.2 μg/g FW when PBST extraction buffer was used, and 729.8 ± 46.2 μg/g FW when Tris-DTT-SDS buffer was used.

### Biochemical characterization of PFA

Unlike other recombinant protein production systems, plants have abundant amounts of starch degrading enzymes which interfere with enzymatic quantitative assays using crude plant extracts. This interference can be reduced by diluting the plant extract if a recombinant protein accumulates to high levels. Indeed, a starch degradation assay using the reducing sugar-DNS method [26] with diluted plant extract can differentiate between wild type and PFA-expressing leaf extract at 16 and 32 fold dilution (Additional file 2: Figure S2). We therefore used extract diluted 20 fold for determining pH and temperature optima for plant-produced PFA.

**Fig. 3:**
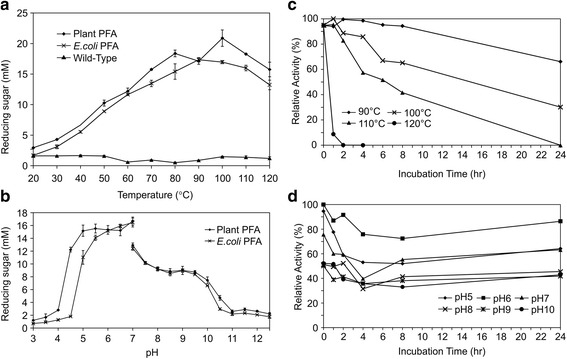
Enzymatic activity and stability of recombinant PFA at different temperatures (**a** and **c**) and pH values (**b** and **d**). **a** The reaction was carried out in acetate buffer at pH 5.5. **b** The reaction was carried out in acetate buffer at pH 3-7, and in phosphate buffer at pH 7-12.5. **c** Plant-made PFA was extracted with reducing buffer, diluted 1/20 in acetate buffer pH 5.5 and incubated at different temperatures. **d** Plant-made PFA was extracted with reducing buffer, diluted 1/20 in buffers with different pH values and incubated at room temperature. The enzymatic assay was carried out at 95 °C

We found that while the optimal temperature for *E.coli*-expressed PFA is 90 °C, there are two peaks for plant expressed PFA at 80 °C and 100 °C (Fig. 3a). It is not clear why there are two peaks. It might be that as the temperature increases, aggregates are dissociated, allowing monomeric PFA better access to the substrate, and leading to an increase in enzymatic activityat 100 °C. Both Dong et al. [6] and Wang et al. [7] reported that *E.coli*-expressed PFA has one peak at 100 °C while our *E.coli*-expressed PFA has a lower optimal temperature of 90 °C. It is possibly due to the fact that our *E.coli*-expressed PFA has extra amino acids at its N-terminal and thus its thermostability is somewhat reduced. Plant-expressed PFA is functional at pH 4.5-10 with an optimal pH of 5-7 and *E.coli*-expressed PFA is functional at pH 5-10 with an optimal pH of 6-7. Therefore, plant-expressed PFA has a slightly broader pH range than *E.coli*-expressed PFA (Fig. 3b). Our results show that at pH 7 PFA exhibits a higher activity in acetate buffer than in phosphate buffer (Fig. 3b), this indicates that the pH is not the sole condition affecting enzymatic activity, and that buffer choice is important for activity assays. Other researchers have consistently used acetate buffer for determining PFA enzymatic activity [1, 3, 6, 7].

Plant-made PFA is highly stable at high temperature like wild-type and *E.coli*-produced PFA [1, 3, 6, 7, 27]. Plant-made PFA was able to sustain 110 °C with no loss of activity for 1 h, and 50% activity after 6 h. At 100 °C, PFA maintained over 60% activity after 8 h of incubation and 30% activity after 24 h incubation. At 90 °C, it barely lost any activity after 8 h of incubation and maintained over 60% activity after 24 h incubation (Fig. 3c). Plant-made PFA is stable at pH 6 for up to 24 h at room temperature. At pH 5 and pH 7, PFA maintained over 60% of its function for up to 24 h at room temperature (Fig. 3d).

We found that plant PFA extracted with buffer containing DTT maintains its thermostability, while Savchenko et al. (2002) showed that DTT significantly reduced the thermostability of PFA at pH 8.5 and 115 °C [21]. The different results could be explained by the different conditions used in the experiments we conducted versus those used by [21]. We extracted PFA from plant tissue with reducing extraction buffer at pH 7.5 and diluted the plant extract in 20 volumes of acetate buffer at pH 5.5 for the activity assay. DTT's reducing activity is only functional at pH above 7, so in our experiment, DTT would have reduced the size of the aggregates, but would not have affected the thermostability in the same way as in the experiment reported by Savchenko et al. [21].

### Purification of recombinant PFA

**Fig. 4:**
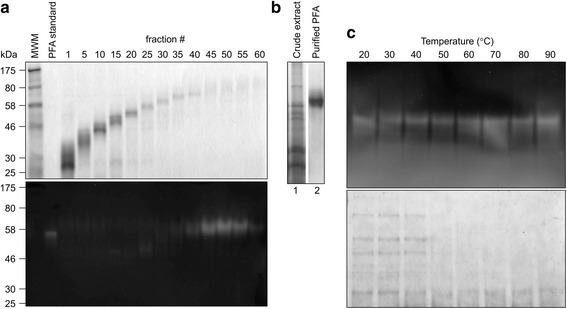
Purification of PFA from plant extract by electrophoresis with a preparative PAGE cell (**a** and **b**) and by heating (**c**). **a** GelCode *blue-stained* PAGE gel (top panel) and zymogram (bottom panel). Ten ml fractions were collected every 8 min for 8 h. Numbers denote the fraction number. Each lane was loaded with 20 μl sample. **b** Gelcode *blue-stained* PAGE loaded with crude extract and purified PFA from pooled fractions 45–55 which contained most PFA and very few other proteins. The pooled sample was concentrated and buffer-exchanged into reducing extraction buffer using a spin column with 10 kDa cut-off pore size. **c** Reduced extract was heated at different temperatures for 5 min and cleared by centrifugation. The supernatant was separated by SDS-PAGE. Top panel: zymogram. Lower panel: GelCode *blue stained* gel

Although plant made PFA is functional in crude plant extract and the industrial use of α-amylase generally doesn't need much purification, purified enzymes are required in pharmaceutical applications [28]. We developed two purification methods for plant-made PFA. In the first method, crude plant extract was fractionated on a preparative PAGE column and the collected fractions were characterized by SDS-PAGE and zymography (Fig. 4a). Fractions 45–55 showed a major band at 70 kDa and were pooled and concentrated; the recovered yield was 0.3 mg PFA/g F.W. at 68.2% purity (Fig. 4b). In the second method, we took advantage of PFA’s thermostability and purified it at large scale simply by heating the plant extract. Using this simple method, contaminating plant proteins were denatured and precipitated at temperatures above 50 °C, while PFA remained in solution and was functional up to 90 °C. The heating step was followed by centrifugation (Fig. 4c).

Considering both enzymatic activity and stability, 95-100 °C and pH 5.5-6.0 are the optimal conditions for plant-made PFA. If one unit is defined as the amount of enzyme to release 1 μmol of reducing sugar in 1 min, using maltose as a standard, the amount of active PFA in plants is 1080 units/g lyophilized leaf tissue from whole leaves containing major veins or 220 units/g FW leaf tissue with the veins removed. It is also worth to mention that the samples used in Figs. 2c and 4c were stored for 5 years at -20 °C in lyophilized tobacco leaf tissue. The fact that PFA is functional after 5 years indicates that plant made PFA could potentially have a long shelf life at room temperature in lyophilized plant leaves

### Field trials

To evaluate the yield potential of PFA in the field, and the feasibility of producing kilogram amounts of PFA, we conducted two field trials in 2009 and 2010. Out of 16 tobacco cultivars that were transformed with PFA [12], we chose 3 lines from each of 6 cultivars, and conducted a field trial in 2009, then we carried one or two lines from each cultivar forward and conducted a second field trial in 2010 (Table 1 and Additional file 3). Both trials were conducted in randomized field plots with 4 replications. The plants were harvested twice over the course of the 4 months growing season, and tobacco biomass and PFA levels were evaluated. We found that the tobacco biomass yield was highest with cultivars 81 V9 and I64 in both years, but that the yield of PFA was variable between 2009 and 2010, and between cultivars. Four cultivars had higher PFA yield in 2010 than in 2009. This may be due to the copy number of the transgene since homozygous plants (*pfa*/*pfa*) were used in 2010 (all T2 seedlings germinated on selective media were green), while in 2009, a mixture of hemizygous (*pfa*/-) and homozygous (*pfa*/*pfa*) plants were used (T1 seedlings germinated on selective media segregated 3 green to 1 white, green seedlings consist of 2 hemizygous:1 homozygous). 81 V9 had good PFA yield in 2009, but in 2010, the yield of PFA decreased to almost inexistent. We suspect that this line underwent silencing of the transgene. The accumulation of PFA reached up to 3.4 g/kg FW (TI 75, 12 F34, 2010), and considering that high density tobacco is planted at 40,000 plants/ha, the potential of PFA production is up to 42.6 kg/ha.

**Table 1:** PFA yield in field grown tobacco

			PFA (g/kg fresh leaf)^a^			Fresh leaf yield (kg/plant)^b^				PFA kg/ha
	Transgenic	Year	first	second		first	second		PFA/plant	if 40,000
cultivar	line		harvest	harvest	average	harvest	harvest	total	g	plant/ha
81 V9	10 F10	2009	0.95 ± 0.34	1.80 ± 0.56	1.38	0.28 ± 0.03	0.31 ± 0.11	0.59	0.81	32.57
	10 F18	2009	0.70 ± 0.16	1.68 ± 0.80	1.19	0.26 ± 0.04	0.28 ± 0.04	0.54	0.64	25.70
		2010	0.21 ± 0.04	0.07 ± 0.01	0.14	0.25 ± 0.10	0.22 ± 0.05	0.47	0.07	2.63
	10 F12	2009	0.55 ± 0.05	1.31 ± 0.11	0.93	0.30 ± 0.05	0.25 ± 0.09	0.55	0.51	20.46
I64	1 F9	2009	1.72 ± 0.26	1.05 ± 0.16	1.38	0.20 ± 0.06	0.27 ± 0.06	0.47	0.65	25.94
	1 F18	2009	1.48 ± 0.49	0.52 ± 0.23	1	0.23 ± 0.02	0.29 ± 0.07	0.52	0.52	20.80
	1 F14	2009	1.31 ± 0.13	1.01 ± 0.23	1.16	0.18 ± 0.06	0.23 ± 0.03	0.41	0.48	19.02
		2010	0.89 ± 0.28	0.38 ± 0.23	0.63	0.25 ± 0.11	0.21 ± 0.04	0.46	0.29	11.59
TI 75	12 F8	2009	1.35 ± 0.15	2.29 ± 0.62	1.82	0.11 ± 0.03	0.14 ± 0.02	0.25	0.46	18.20
	12 F34	2009	0.85 ± 0.26	1.01 ± 0.17	0.93	0.11 ± 0.02	0.18 ± 0.02	0.29	0.27	10.79
		2010	2.39 ± 0.51	4.49 ± 0.47	3.44	0.19 ± 0.04	0.12 ± 0.01	0.31	1.07	42.66
	12 F35	2009	0.40 ± 0.13	0.90 ± 0.23	0.65	0.14 ± 0.02	0.21 ± 0.07	0.35	0.23	9.10
Con.	7 F8	2009	0.53 ± 0.13	1.28 ± 0.24	0.91	0.25 ± 0.03	0.22 ± 0.01	0.47	0.43	17.11
Havana 38		2010	1.97 ± 0.59	1.24 ± 0.31	1.61	0.19 ± 0.02	0.15 ± 0.04	0.34	0.55	21.90
	7 F13	2009	0.67 ± 0.10	1.25 ± 0.11	0.96	0.17 ± 0.03	0.16 ± 0.05	0.33	0.32	12.67
	7 F15	2009	0.47 ± 0.26	0.40 ± 0.03	0.43	0.32 ± 0.05	0.22 ± 0.02	0.54	0.23	9.29
TI 95	3 F5	2009	0.83 ± 0.35	0.99 ± 0.12	0.91	0.14 ± 0.01	0.20 ± 0.05	0.34	0.31	12.38
		2010	2.47 ± 0.44	0.41 ± 0.13	1.44	0.14 ± 0.03	0.15 ± 0.04	0.29	0.42	16.70
	3 F4	2009	1.48 ± 0.32	0.62 ± 0.20	1.05	0.15 ± 0.03	0.15 ± 0.06	0.3	0.32	12.60
	3 F30	2009	0.34 ± 0.07	0.45 ± 0.06	0.4	0.17 ± 0.04	0.20 ± 0.02	0.37	0.15	5.92
Little	9 F28	2009	1.10 ± 0.14	0.52 ± 0.30	0.81	0.17 ± 0.01	0.18 ± 0.06	0.35	0.28	11.34
Crittenden		2010	2.09 ± 0.62	2.67 ± 0.92	2.38	0.22 ± 0.06	0.16 ± 0.02	0.38	0.90	36.18
	9 F20	2009	0.80 ± 0.07	0.55 ± 0.01	0.68	0.17 ± 0.03	0.19 ± 0.04	0.36	0.24	9.79
		2010	2.31 ± 0.37	0.75 ± 0.49	1.53	0.25 ± 0.08	0.18 ± 0.04	0.43	0.66	26.32
	9 F26	2009	0.18 ± 0.02	0.62 ± 0.43	0.4	0.18 ± 0.05	0.20 ± 0.03	0.38	0.15	6.08

a: The data are expressed as the mean ± SD from four experimental plots

b: the data are expressed as the mean ± SD from 40 (2009 first harvest), 12 (2009 s harvest) and 20 (2010 first and second harvest) plants

## Conclusions

We conclude that α-amylase from *P. furiosus* (PFA) can be expressed in tobacco and accumulates as insoluble aggregates which can be solubilized with reducing extraction buffer. The size of the aggregates can be larger than 1000 kDa. Plant-made PFA is functional without requiring refolding procedures and therefore has a potential to be applied as plant tissue without purification in some applications. Plant-made PFA in lyophilized plant tissue also has the potential for long shelf life at room temperature. While tobacco represents an alternative production system for PFA, scaling-up production should be done on marginal land unsuitable for food crop production to avoid competing with food production on fertile agricultural land. Although we were able to produce active PFA at a yield of 42.6 kg/ha tobacco, its economic benefit over bacteria still needs to be further evaluated. Improvement using fusion tags could also be explored in future work to further increase the yield of plant made PFA and make it more economical in industrial production.

## Methods

### Expression and purification of PFA in *E.coli* and antibody production

The *pfa* gene was amplified by PCR using *P. furiosus* genomic DNA (ATCC# 43587D-5) as template (Genebank accession number AF001268) [6] without its signal peptide sequence [7]. *EcoR* I and *Hind* III sites were introduced into the forward and reverse primers (forward primer: GAGGAATTCAAATACTTGGAGCTTGAAGAGGG; reverse primer: ATCAAGCTTTCACCCAACACCACAATAACTCCAT) to facilitate cloning into *E.coli* expression vector PET 32 (Novagen-EMD Millipore, Etobicoke, ON, Canada). *E.coli* BL21 (DE3+) (Novagen-EMD Millipore) was used for expression, and inclusion bodies were solubilized and refolded using a protein refolding kit from Novagen-EMD Millipore. The refolded PFA was then concentrated with Centriprep spin column (EMD-MilliPore) and purified by PAGE using a PrepCell apparatus (BioRad, Mississauge, Ontario, Canada) with 1x SDS gel running buffer as elution buffer. PAGE-purified *E.coli* PFA was dialyzed with PBS and quantified on a PAGE stained with GelCode Blue protein stain (Fisher Scientific Canada, Ottawa, Ontario, Canada) following the protocol from the manufacturer. Bovine Serum Albumin (BSA) solution with known concentration was used as standard. Purified *E.coli* made PFA was used as a positive control in protein analysis and quantification of plant made PFA, and as antigen to produce polyclonal anti-PFA antibody. Antibody was produced in two rabbits by the Animal Care and Veterinary Service at the University of Western Ontario (London, Ontario, Canada). The serum was collected 8 weeks after first injection. The unpurified serum was used as anti-PFA antibody in Western hybridization analysis.

### Constructs for plant transformation

The *pfa* gene was codon optimised for tobacco codon-usage [14] and was synthesized by GeneArt-Life Technologies Inc. (Burlington, Ontario, Canada). Both wild type and codon-optimized *pfa* genes were cloned into pCaMterX binary vector [17] under the control of double 35S promoter [29] and Nos terminator [30]. Tobacco secretory signal peptide Pr1b [31] was fused to the N-terminal of the gene and StrepII tag [32] and the ER retrieval tetrapeptide KDEL [33] were fused to the C-terminus of the gene

### Transient expression analysis and stable transformation

The plasmids carrying *pfa* constructs were transformed into *Agrobacterium tumefaciens* strain EHA105. Transient expression was carried out using a protocol reported previously [34]. Stable transgenic lines were generated by using the protocol from Horsch et al. [35].

### Extraction and detection of recombinant PFA

Leaf discs were collected using 0.85 cm diameter cork borer (size 5). Eight leaf discs were collected from 8 different leaves from each transgenic plant and put into a 2 ml tube with three 2.3 mm zirconia silica beads (Fisher Scientific, Ottawa, Canada). The samples were frozen in liquid nitrogen and pulverized with a TissueLyser (Qiagen, Toronto, Ontario, Canada). Plant protein was extracted with either PBS-Tween based extraction buffer (PBS pH 7.4 plus 0.1% tween 20, 2% PVPP, 1 mM EDTA, 1 mM PMSF, 1 ug/ml leupeptin and 100 mM ascorbic acid), or reducing extraction buffer (50 mM Tris, pH 8.0, 1% SDS and 20 mM DTT). Each pulverized leaf sample was extracted with 0.4 ml buffer by mixing with a vortex mixer and cleared by centrifugation at 20,000 x *g* for 15 min at 4 °C (PBST extraction buffer) or 5 min at 22 °C (reducing extraction buffer). Plant proteins were denatured by heating to 95 °C for 10 min in SDS loading buffer (60 mM Tris, pH 8.0, 1% SDS, 20 mM DTT, 10% glycerol and 0.01% phenol red) and separated by 10% SDS PAGE at 100 V for 2 h. Separated proteins were transferred to polyvinylidene difluoride (PVDF) membranes and blocked overnight in 5% (w/v) skim milk powder in PBST (phosphate-buffered saline with 0.1% Tween 20). Membranes were incubated in a 1/2000 dilution of anti-PFA antibody for 1 h at room temperature. After washing with PBST, membranes were incubated in 1/5000 dilution of horse radish peroxidase (HRP)-conjugated goat anti-rabbit secondary antibody (Bio-Rad) for 1 h at room temperature. The washed membranes were detected with ECL Western Blotting Detection Reagents (GE Healthcare, Baie d´Urfé, Québec, Canada), and autoradiography. PFA was quantified by image densitometry with TotalLab Quant software (Nonlinear Dynamics, Durham, NC, USA). Known amount of purified PFA expressed in *E.coli* was used as standard for quantification.

### Zymography of amylase activity

To determine the activity of plant-produced PFA, 10% SDS or native polyacrylamide gels containing separated plant proteins were incubated in acetate buffer (50 mM sodium acetate, pH 5.5) for 15 min at 40 °C, followed by incubation in 1% soluble corn starch (Sigma-Aldrich, Oakville, Ontario, Canada) dissolved in acetate buffer for 30 min at 95 °C. The gels were briefly rinsed with water and stained with iodine solution (10 mM iodine, 100 mM potassium iodine) until color developed. The stained gels were rinsed with water and imaged by scanning with a scanner or by photographing with a camera.

### Purification of plant-made PFA from transgenic tobacco

To purify plant made PFA from tobacco with prep scale PAGE, the leaf tissue of PFA transgenic tobacco was ground in reducing extraction buffer with the ratio of 1 g leaf tissue to 3 ml buffer in a Waring blender for 20 s at low speed followed by 3 × 20 s at high speed. The homogenized leaf tissue then was cleared by centrifugation at 20,000 x *g* for 15 min and the supernatant was collected as plant extract. The plant extract was mixed with 5 x SDS sample loading buffer (0.3 M Tris-HCl, pH 8.0, 5% SDS, 50% glycerol, 100 mM DTT, 0.05% w/v phenol red) and denatured by boiling for 10 min. The proteins were separated by PAGE using a PrepCell apparatus (BioRad) with 1x SDS gel running buffer as elution buffer. Proteins were eluted in 5–10 ml fractions and subjected to zymogram analysis. The fractions containing PFA were pooled, concentrated and buffer exchanged into reducing extraction buffer using a Centriprep spin column (EMD-MilliPore) with 10 kDa cut-off pore size. The concentration of the purified PFA was determined by zymogram of starch degradation assay with purified *E.coli*-made PFA as standard and the purity was determined by PAGE followed by staining the gel with GelCode Blue protein stain (Fisher Scientific). The concentration of total protein in reducing extraction buffer was determined with *RC DC* Protein Assay reagent (BioRad) using the protocol provided by the manufacturer.

### Enzymatic activity determination

The starch degradation activity of the recombinant PFA was quantitated with dinitrosalicylic acid (DNS) starch degradation assay [26] using maltose as standard. The plant extract with reducing extraction buffer from 20 mg powdered freeze dried whole leaf was diluted 1 in 20 in either acetate buffer (50 mM sodium acetate) or phosphate buffer (100 mM) with different pH according to experiments. For optimal temperature assessment, acetate buffer with pH 5.5 was used to dilute the plant extract. The diluted plant extract was then mixed with 1% water soluble starch in acetate or phosphate buffer at 1 to 1 ratio and incubated for 15 min at designed temperature. After incubation, the samples were cooled on ice to stop reaction. Color development was carried out by mixing the samples at 1 to 1 ratio (v/v) with Color Reagent Solution prepared by dissolving 12 g of sodium potassium tartrate, tetrahydrate in 8.0 ml of 2 M NaOH (Solution A), and 0.46 g DNS in 20 ml water (Solution B). Solution A and Solution B were then combined and topped with water to 40 ml to make the Color Reagent solution. The color was developed by incubation at 95 °C for 15 min. The product of the color development reaction was then diluted with water at 1 to 1 ratio in a 96-well microplate. The concentration of reducing sugar was determined by reading the plate at wavelength of 540 nm.

### Field trials

Two confined research field trials were conducted at our research farm in Delhi, Ontario, in 2009 and 2010. In 2009, three selected independent transgenic lines of 6 tobacco cultivars (3 lines X 6 cultivars for a total of 18 treatments) were evaluated in randomized field plots with 4 replications. The plants were self-pollinized T0 progeny. The lines were chosen according to their PFA accumulation level and segregation on Kanamycin selective medium. Lines showing single insertion segregation (3:1) were used, except one line in TI 75 with a double insertion (line 12 F8, Table 1). The experiment was repeated in 2010 with T2 lines homozygous for the *pfa* gene. One to two lines from each cultivar having the highest PFA accumulation and biomass yield in the 2009 field trial was used. Homozygozity was determined by testing the resistance of seedlings to kanamycin on selective medium. The lines with 100% green seedlings on kanamycin selective medium were considered to be homozygous for *pfa*.

In both years, plots consisted of 4 rows X 8 m long, spaced 0.3 m apart, with plants spaced 0.5 m apart in each row for a total of about 17 plants per row. The middle 2 rows contained GMO plants and the outer 2 rows non-GMO plants. Fertilizer (50 kg N + 56 kg P_2_O_5_ + 150 kg K_2_O/ha) was incorporated into the soil prior to transplanting. All field data were collected from the middle 2 rows.

In early August, about 60 days after transplanting to the field, the plants were harvested. All plants were cut about 0.2 m above the soil level. Ten plants per plot were used for data collection. For this, plants were separated into leaf and stalk components and weighed to determine relative yields of the first harvest. After weighing, sub-samples with about 0.1 g of leaf tissue were taken for PFA yield analysis. An additional 50 kg N/ha was applied by hand and incorporated into the soil between the plot rows. Suckers sprouting from the residual stalk and root system (1–5 per plant) were allowed to grow until they were close to flowering. In late September, a second harvest was made following procedures similar to the first harvest, relative yields were determined and samples taken for PFA yield analyses.

## Additional files

Additional file 1: Figure S1. Optimization of protein extraction buffer. Zymograms of starch degradation with recombinant PFA. The same amount of plant extract was loaded onto each lane. a) Plant-made PFA extracted with different buffers. The average accumulation level with PBST is 66.3 ± 9.2 mg/g FW, with Tris-DTT-SDS is 729.8 ± 46.2 mg/g FW and with Tris-SDS is 652.0 ± 57.6 mg/g FW. b) PFA extracted in reducing buffer with and without SDS. c) PFA extracted with different concentration of SDS. d) The stability of active PFA with and without DTT. Plant proteins were extracted with Tris-DTT-SDS and diluted 10 fold in Tris-SDS buffer with or without DTT and incubated at room temperature for 2.5 h. (TIF 871 kb)

Additional file 2: Figure S2. Diluting plant extracts reduces interference of endogenous starch degradation enzymes. The error bars represent the standard deviation of three technical replicates. (TIF 246 kb)

Additional file 3: Field trial raw data. (XLSX 46 kb)

## Abbreviations

EDTA: ethylenediaminetetraacetic acid
ER: endoplasmic reticulum
PAGE: polyacrylamide gel electrophoresis
PBS: phosphate-buffered saline
PBS-T: phosphate-buffered saline-tween 20
PFA: *Pyrococcus furiosus* amylase
PMSF: phenylmethylsulfonyl fluoride
PVPP: polyvinylpolypyrrolidone
SDS: sodium dodecyl sulfate
