# Supplementary figures and images for: A hyper-thermostable α-amylase from Pyrococcus furiosus accumulates in Nicotiana tabacum as functional aggregates
Source: BMC Biotechnol. 2017 Jun 19;17:53. doi: 10.1186/s12896-017-0372-3 (PMC5477289; doi:10.1186/s12896-017-0372-3)

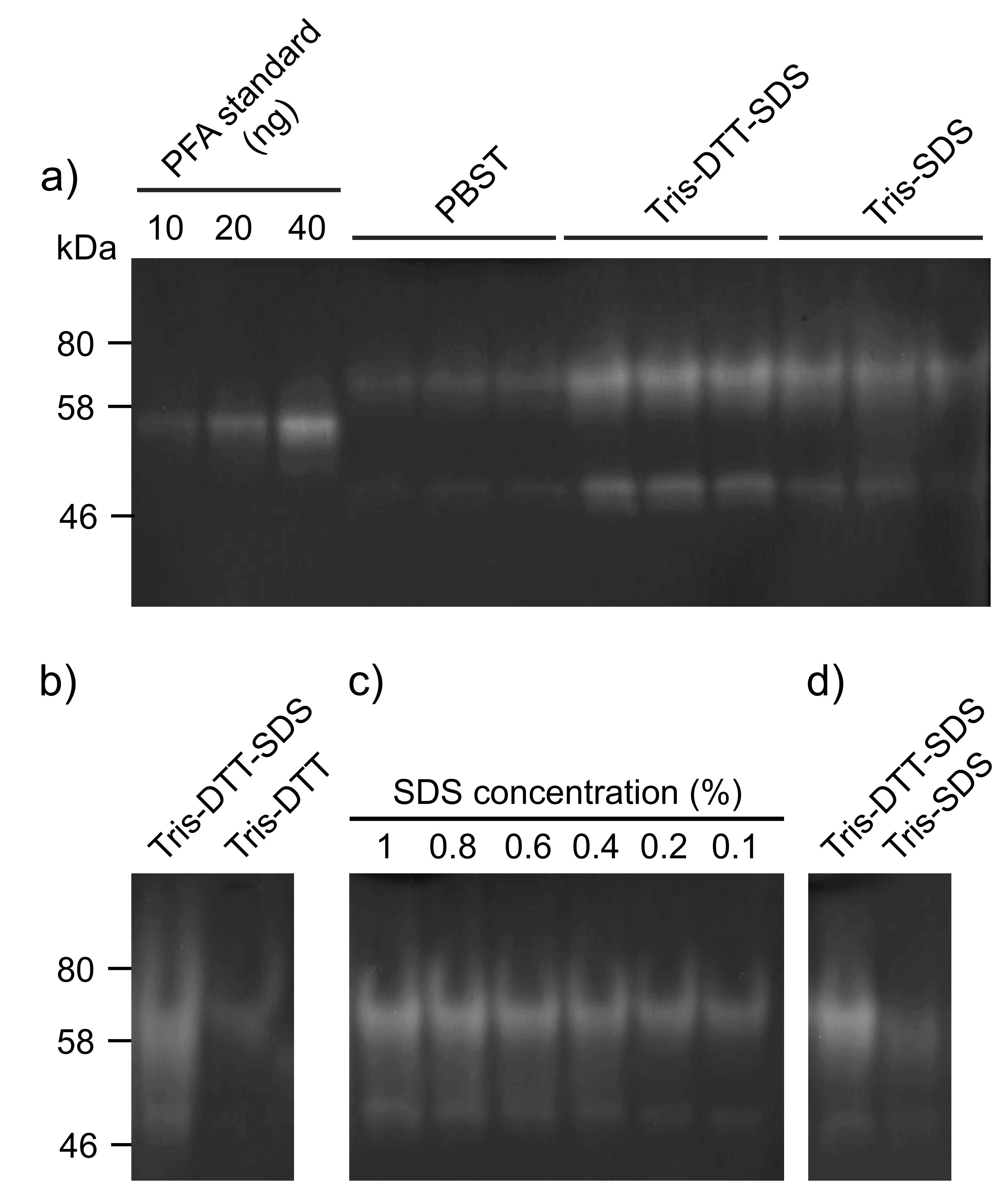

Supplement: Supplementary file 1 — Optimization of protein extraction buffer. Zymograms of starch degradation with recombinant PFA. The same amount of plant extract was loaded onto each lane. a) Plant-made PFA extracted with different buffers. The average accumulation level with PBST is 66.3 ± 9.2 mg/g FW, with Tris-DTT-SDS is 729.8 ± 46.2 mg/g FW and with Tris-SDS is 652.0 ± 57.6 mg/g FW. b) PFA extracted in reducing buffer with and without SDS. c) PFA extracted with different concentration of SDS. d) The stability of active PFA with and without DTT. Plant proteins were extracted with Tris-DTT-SDS and diluted 10 fold in Tris-SDS buffer with or without DTT and incubated at room temperature for 2.5 h. (TIF 871 kb) [file 12896_2017_372_MOESM1_ESM.tif]

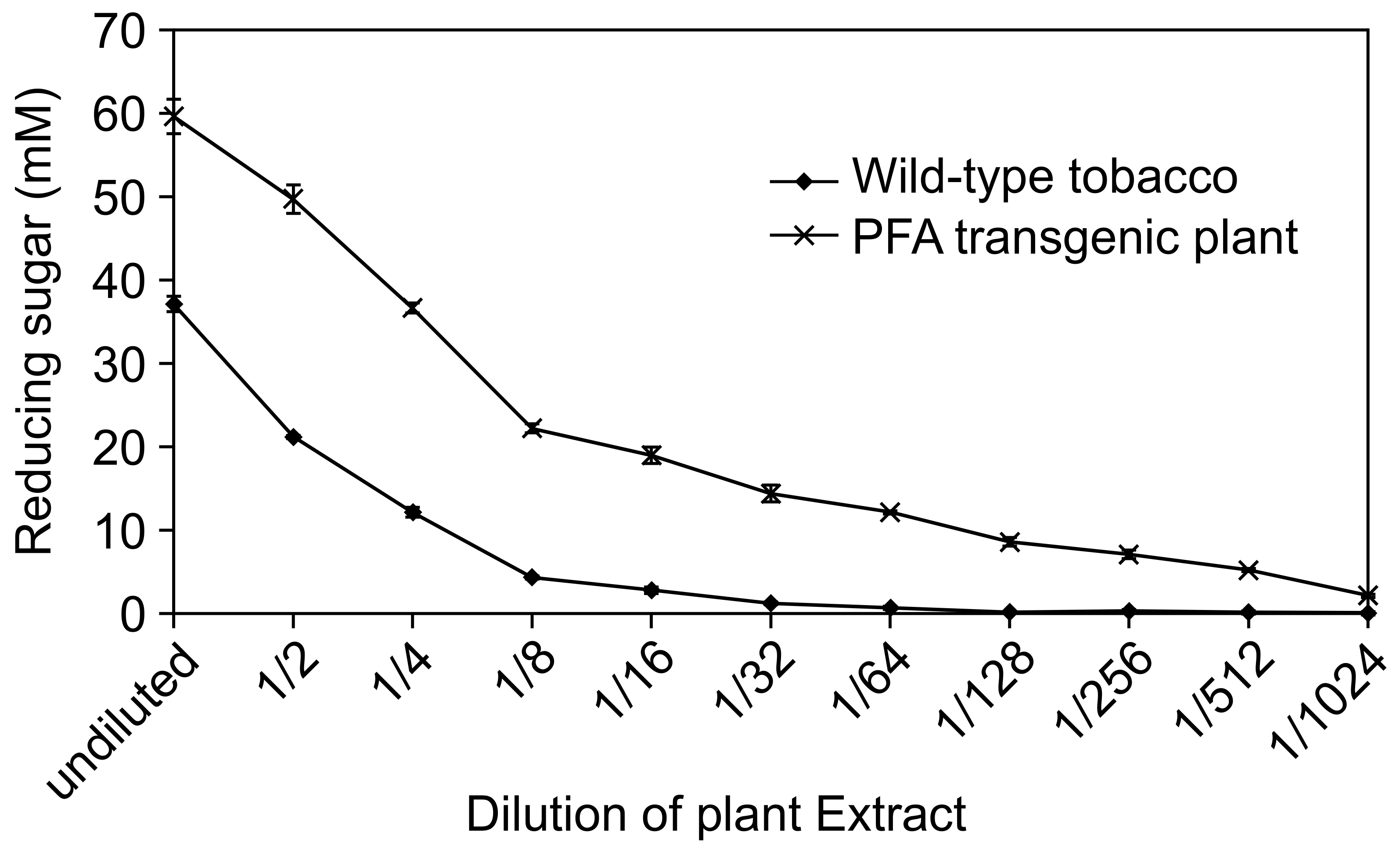

Supplement: Supplementary file 2 — Diluting plant extracts reduces interference of endogenous starch degradation enzymes. The error bars represent the standard deviation of three technical replicates. (TIF 246 kb) [file 12896_2017_372_MOESM2_ESM.tif]
